# The Forty-First Annual Course of Instruction in the Baltimore College of Dental Surgery

**Published:** 1880-10

**Authors:** 


### EDITORIAL, ETC.

---

*The Forty-first Annual Course of Instruction in the Baltimore College of Dental Surgery,* began under the most favorable auspices on the 14th of October, 1880. The students in attendance upon the opening lecture of the Course numbered over fifty, more than were ever before present at such an early period in the Annual Session of the College during an existence of over forty years. This number has been considerably increased since the fourteenth inst., and there is every reason to conclude that the class of 1880–81, will be the largest the College has ever known.

The desire to vote at the coming Presidential election has delayed the arrival of quite a number, whose appearance will considerably add to the strength of the present class.

This unprecedented prosperity of their *Alma Mater* will be hailed with pleasure by every Alumnus who feels interested in the welfare of the College.

The opening lecture of the course was delivered on the 14th inst. by Prof. F. J. S. Gorgas, and embraced a history of the causes which so long delayed progress towards attaining a correct insight into the pathology of the teeth, when every other department of medical science was receiving the attention it merited. The extensive range of affiliation which dentistry in its completeness possesses, was commented upon, and the relations to other sciences referred to. The mechanical skill necessary to ensure perfection in the dental art when exercised for a noble purpose was regarded as never degrading, but ennobling; the certainty and success thus attained not being equaled by even medicine, although a much older art.

The necessity for earnest and willing workers in the profession was argued, on the ground that a life of usefulness is sweetened by duty and rewarded by honor.

At the close of the lecture a meeting of the students was held, and a committee appointed, who addressed the following communication to Prof. Gorgas:

BALTIMORE COLLEGE OF DENTAL SURGERY OCT. 14, 1880.

DR. GORGAS,—*Dear Sir—*

At a meeting of the students of the Baltimore College of Dental Surgery it was unanimously and enthusiastically decided that a committee should wait upon you and ask the privilege of publishing in pamphlet form, your lecture delivered before the class this morning, believing the most excellent advice contained therein to be of great service to us in our future career, and desiring always to keep it before us.

We remain very respectfully yours,

EFF. WAGNER, ALA.

*President.*

W. G. FOSTER, MD.

H. S. PITTS, VA.

B. S. SMITH, JR., MD.

*Committee.*

Last year the faculty of the Baltimore College adopted the beneficiary system, which enables one deserving young man on the recommendation of his State Dental Association to enter the college on the payment of but half of the tuition fees. During the year six State Dental Associations sent such students, and this year twelve State Dental Associations have conferred this privilege on deserving young men, viz.—New York, Massachusetts, North Carolina, South Carolina, Georgia, New Jersey, Connecticut, Maryland and District of Columbia, Pennsylvania, Lousiana and Alabama.

Four applications for such a scholarship have been received from young men in Virginia, and when they were referred to the President of the Virginia State Dental Association, the answer returned was that this Association had determined to recommend no applicants for the beneficiary system. Why the Virginia Association should adopt such action to the detriment of worthy young men, is beyond our comprehension, especially when all other State Dental Societies approve of the beneficiary system and have shown a willingness to assist in promoting its object.

---

### BIBLIOGRAPHICAL.

